# Wiederanstieg der Januskinaseinhibitor-Neuverordnungen: Eine Analyse der deutschen RHADAR-Datenbank

**DOI:** 10.1007/s00393-025-01728-7

**Published:** 2025-09-23

**Authors:** Patrick-Pascal Strunz, Linus Maximilian Risser, Matthias Englbrecht, Torsten Witte, Matthias Fröhlich, Marc Schmalzing, Michael Gernert, Johannes Heck, Peter Bartz-Bazzanella, Cay von der Decken, Kirsten Karberg, Georg Gauler, Susanna Späthling-Mestekemper, Christoph Kuhn, Wolfgang Vorbrüggen, Martin Welcker, Stefan Kleinert

**Affiliations:** 1 Medizinische Klinik II, Rheumatologie/Immunologie, Universitätsklinikum Würzburg, Oberdürrbacher Str. 6, 97080 Würzburg, Deutschland; 2 Klinik für Rheumatologie & Immunologie, Medizinische Hochschule Hannover, Hannover, Deutschland; 3 Greven, Deutschland; 4 Institut für Klinische Pharmakologie, Medizinische Hochschule Hannover, Hannover, Deutschland; 5 Klinik für Internistische Rheumatologie, Rhein-Maas-Klinikum, Würselen, Deutschland; 6 Medizinisches Versorgungszentrum, Stolberg, Deutschland; 7 RHADAR – RheumaDatenRheport GbR, Planegg, Deutschland; 8 Verein zur Förderung der Rheumatologie e. V., Würselen, Deutschland; 9 Rheumatologisches Versorgungszentrum Steglitz, Berlin, Deutschland; 10 Rheumatologische Praxis Osnabrück, Osnabrück, Deutschland; 11 Rheumapraxis München, München, Deutschland; 12 Praxis für Rheumatologie, Karlsruhe, Deutschland; 13 Medizinisches Versorgungszentrum für Rheumatologie Dr. M. Welcker GmbH, Planegg, Deutschland; 14 Praxisgemeinschaft Rheumatologie-Nephrologie, Erlangen, Deutschland

**Keywords:** Oral Surveillance-Studie, Sicherheitsempfehlungen, Versorgungsforschung, Pharmakovigilanz, Rheumatoide Arthritis, Oral Surveillance Study, Safety recommendations, Real world evidence, Pharmacovigilance, Rheumatoid arthritis

## Abstract

**Hintergrund:**

Durch behördliche Sicherheitsempfehlungen zu Januskinaseinhibitoren (JAKi) kam es zu einem deutlichen Rückgang der Neuverordnungen in Deutschland. In der Zwischenzeit erschienen auch differenzierte Handlungsempfehlungen von Fachgesellschaften.

**Ziel der Arbeit:**

Es sollte mittels des RHADAR-Registers untersucht werden, ob sich die Verordnungen in der Folge wieder erholten.

**Material und Methoden:**

Retrospektive Analyse der Neuverordnungen von JAKi, TNF-Inhibitoren (TNFi) und IL-6-Rezeptor-Inhibitoren (IL-6Ri) bei Patient:innen mit rheumatoider Arthritis zwischen dem 01.04.2020 und dem 30.09.2024. Primärer Endpunkt war der relative Anteil der JAKi-Neuverordnungen an allen Neuverordnungen dieser 3 Substanzklassen pro Halbjahr. Zusätzlich wurde die Therapielinie bei JAKi erfasst.

**Ergebnisse:**

Insgesamt wurden 3492 Behandlungsneubeginne im Register im Zeitraum erfasst (TNFi: *n* = 1770, 50,7 %, JAKi: *n* = 1269, 36,3 %, IL-6Ri: *n* = 453, 13,0 %). Der Anteil von JAKi stieg zunächst von 29,5 % (Quartal [Q] 2–Q3/2020) auf 46,9 % (Q2–Q3/2021) und fiel nach den behördlichen Sicherheitsempfehlungen auf 24,4 % (Q2–Q3/2023). Seit Q4/2023 ist ein Wiederanstieg zu verzeichnen (32,8 %), jedoch ohne das Vorniveau wieder zu erreichen. JAKi wurden zunehmend aus den vorderen in die fortgeschrittenen Therapielinien verdrängt (≥ 3. Linie).

**Schlussfolgerung:**

Nach dem Rückgang der JAKi-Neuverordnungen durch die EMA-Sicherheitshinweise kam es zuletzt zu einem Wiederanstieg. JAKi werden seither bevorzugt in fortgeschrittenen Therapielinien eingesetzt, was auf einen differenzierteren Einsatz dieser Substanzklasse hindeutet.

## Abstract (en)

**Background:**

Regulatory safety recommendations regarding Janus kinase inhibitors (JAKi) led to a marked decline in new prescriptions in Germany. In the meantime, professional medical societies have also issued differentiated recommendations.

**Objectives:**

The aim was to investigate the RHADAR (RheumaDatenRhePort) registry to check whether JAKi prescriptions recovered thereafter.

**Materials and methods:**

A retrospective analysis of new prescriptions for JAKi, tumor necrosis factor inhibitors (TNFi), and interleukin‑6 receptor inhibitors (IL-6Ri) in patients with rheumatoid arthritis between 1 April 2020, and 30 September 2024. The primary endpoint was the proportion of JAKi among all new prescriptions of these three drug classes per half-year. The treatment line of JAKi use was additionally assessed.

**Results:**

A total of 3492 treatment initiations were recorded in the registry during the observation period (TNFi: *n* = 1770, 50.7%, JAKi: *n* = 1269, 36.3%, IL-6Ri: *n* = 453, 13.0%). The proportion of JAKi initially rose from 29.5% (Q2–Q3/2020) to 46.9% (Q2–Q3/2021), followed by a decline to 24.4% (Q2–Q3/2023) after the regulatory safety communications. Since Q4/2023, a renewed increase has been observed (32.8%), though not yet reaching previous peak levels. JAKi were increasingly shifted from early to advanced treatment lines (≥ 3rd line).

**Conclusion:**

Following the decline in JAKi prescriptions due to European Medicines Agency safety recommendations, a renewed increase has been observed. Since then, JAKi have been predominantly used in later treatment lines, indicating a more differentiated approach to this drug class.

Neben Leitlinien können auch regulatorische Maßnahmen von Behörden einen Einfluss auf das Behandlungs- und Verschreibungsverhalten von Ärzt:innen haben. Ein Beispiel aus jüngster Zeit sind hier die Sicherheitsempfehlungen der Europäischen Arzneimittel-Agentur (EMA) zu Januskinaseinhibitoren (JAKi) [1]. Dieser Artikel soll prägnant den Einfluss verschiedener Empfehlungen auf das JAKi-Verordnungsverhalten bei der rheumatoiden Arthritis (RA) in Deutschland skizzieren:

Durch die Veröffentlichung der ORAL Surveillance-Studie [2] und der nachfolgenden restriktiven Sicherheitsempfehlung der EMA bezüglich JAKi kam es bei behandelnden Ärzt:innen zu einem veränderten Verschreibungsverhalten von JAKi für die RA in Deutschland: In einer ersten Analyse der RHADAR-Datenbank über den Zeitraum vom 01.04.2020 bis zum 23.09.2023 wurde gezeigt, dass in der Folge dieser behördlichen Maßnahmen die Neuverordnungen von JAKi in Deutschland zugunsten anderer zielgerichteter Therapien mit „disease modifying antirheumatic drugs“ (DMARDs) abnahmen und dass JAKi aus den vorderen Therapielinien zunehmend in die hinteren Therapielinien verdrängt wurden [1]. In einer Follow-up-Analyse der RHADAR-Datenbank mit der gleichen Methodik wie in [1] sollte nun analysiert werden, wie sich durch differenzierte Empfehlungen von Fachgesellschaften wie der deutschen Gesellschaft für Rheumatologie und Klinische Immunologie (DGRh) [3] das Verordnungsverhalten von JAKi bei der RA im Analysezeitraum vom 23.09.2023 bis zum 30.09.2024 weiterentwickelt hat.

Im Zeitraum vom 01.04.2020 bis zum 30.09.2024 wurden in der RHADAR-Datenbank insgesamt 3492 Behandlungsneubeginne mit einem JAKi, TNFi oder IL-6-Ri erfasst. Diese 3 DMARDs stellten in der RHADAR-Datenbank die mit Abstand häufigsten Klassen an zielgerichteten Therapien dar. Der Großteil entfiel dabei auf TNFi (1770 Fälle, entsprechend 50,7 %), gefolgt von JAKi mit 1296 Fällen (36,3 %) und schließlich IL-6Ri mit 453 Fällen (13,0 %). Die demografischen Merkmale sowie das Komorbiditätsprofil der behandelten Patient:innen unterschieden sich nur geringfügig zwischen den Wirkstoffgruppen (Tab. 1). Patient:innen, die mit einem TNFi behandelt wurden, waren im Durchschnitt etwas jünger (57,7 Jahre, SD 15,3) als jene unter JAKi- (60,2 Jahre, SD 13,2) oder IL-6Ri-Therapie (61,7 Jahre, SD 13,8). Zudem war die Krankheitsdauer bei TNFi-Neubeginner:innen mit durchschnittlich 11,9 Jahren kürzer als bei den übrigen Gruppen (z. B. 14,0 Jahre bei JAKi) (Tab. 1).

**Tab. 1:** Patient:innencharakteristika bei Therapiestart

	JAKi	TNFi	IL-6Ri
*Anzahl neuer Therapien n (% von allen Therapie)*	1269 (36,3 %)	1770 (50,7 %)	453 (13 %)
*Frauen n, % („missing“ n, %)*	1033, 79,3 % (0, 0 %)	1396, 78,9 % (0, 0 %)	359, 79,2 % (0, 0 %)
*Alter in Jahren, SD („missing“ n, %)*	60,2, SD 13,2 (0, 0 %)	57,7, SD 15,3 (0, 0 %)	61,7, SD 13,8 (0, 0 %)
*Krankheitsdauer in Jahren, SD („missing“ n, %)*	14,0, SD 10,2 (106, 8,4 %)	11,9, SD 10,2 (223, 12,6 %)	12,2, SD 9,5 (54, 11,9 %)
*Seropositivität n/% (valide n/N, %)*	818, 64,5 % (0, 0 %)	1033, 58,4 % (0, 0 %)	258, 57,0 % (0, 0 %)
*DAS28 („missing“ n, %)*	3,8 (443, 34,9 %)	3,4 (710, 40,0 %)	3,5 (171, 37,7 %)
*SDAI („missing“ n, %)*	18,1 (563, 44,4 %)	14,6 (926, 52,3 %)	15,7 (220, 48,6 %)
*CDAI („missing“ n, %)*	17,0 (563, 44,4 %)	13,7 (925, 52,3 %)	14,8 (219, 48,3 %)
**Ausgewählte Begleiterkrankungen in der Vorgeschichte *n* (%)**			
*Fehlende Daten/keine Dokumentation*	32 (3,4 %)	70 (6,1 %)	11 (2,7 %)
*Keine der ausgewählten Begleiterkrankung dokumentiert*	11 (1,2 %)	32 (2,8 %)	4 (1,0 %)
*Diabetes*	76 (8,0 %)	94 (8,1 %)	38 (9,4 %)
*Hypertonie*	372 (39,5 %)	454 (39,3 %)	156 (38,3 %)
*Venenthrombose*	11 (1,2 %)	34 (3,0 %)	11 (2,7 %)
*Lungenembolie*	8 (0,8 %)	18 (1,6 %)	6 (1,5 %)
*Koronare Herzerkrankung*	110 (11,7 %)	116 (10,1 %)	51 (12,7 %)
*Akuter Myokardinfarkt*	2 (0,2 %)	2 (0,2 %)	1 (0,2 %)
*Schlaganfall*	24 (2,5 %)	26 (2,3 %)	8 (2,0 %)
*Dyslipidämie*	193 (20,5 %)	212 (18,4 %)	67 (16,7 %)
*Nikotinabusus*	108 (8,5 %)	143 (8,1 %)	34 (7,5 %)
*Malignome*	67 (5,3 %)	76 (4,3 %)	30 (6,6 %)

*CDAI* Clinical Disease Activity Index, *DAS28* Disease Activity Score 28, *JAKi* Januskinaseinhibitoren, *TNFi* Tumornekrosefaktorinhibitoren, *IL-6Ri* Interleukin-6-Rezeptor-Inhibitoren, *SD* Standardabweichung, *SDAI* Simplified Disease Activity Index

## JAKi-Neuverordnungen seit Ende 2023

**Abb. 1:**
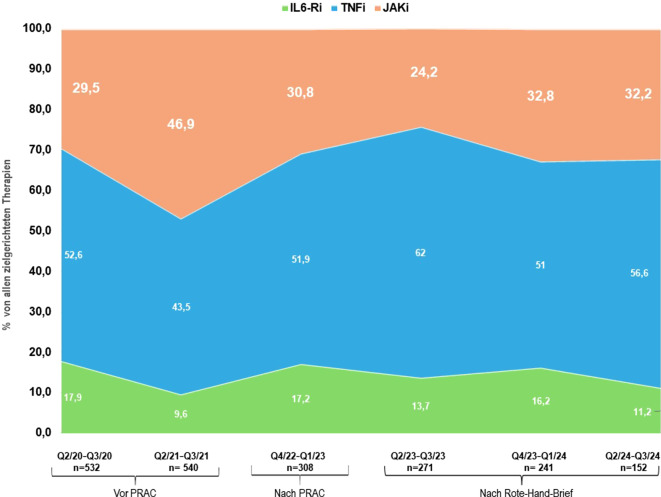
Veränderungen des Anteils von JAKi, TNFi und IL-6Ri an den Neuverordnungen im jeweiligen Halbjahr. *IL-6Ri* Interleukin-6-Rezeptor-Inhibitor, *JAKi* Januskinaseinhibitor, *PRAC* Pharmacovigilance Risk Assessment Comittee recommendations, *Q* Quartal, *TNFi* Tumornekrosefaktorinhibitor

Wie bereits in der vorherigen Studie gezeigt, waren die JAKi-Neuverordnungen signifikant von 29,5 % in dem Halbjahr Quartal (Q)2/2020–Q3/2020 auf 46,9 % im Halbjahr Q2/2021–Q3/2021 angestiegen (Abb. 1; [1]). In der Folge kam es zu einem Rückgang der neu begonnenen JAKi-Therapien: Der Effekt zeigte sich besonders nach den Pharmacovigilance Risk Assessment Committee(PRAC)-Empfehlungen (30,8 %; Q4/2022–Q1/2023) und nach Veröffentlichung des Rote-Hand-Briefs (24,4 %; Q2/2023–Q3/2023; Abb. 1). Ab dem Q4/2023–Q1/2024 begannen die Neuverordnungszahlen wieder anzusteigen (32,8 %), erreichten aber noch nicht wieder das Ausgangsniveau. Der Anteil neu initiierter TNFi-Therapien verhielt sich kompensatorisch umgekehrt und damit indirekt proportional zu den JAKi-Neuverordnungen. Der Anteil neu begonnener Therapien mit IL-6Ri blieb über alle Phasen hinweg relativ stabil (Abb. 1).

**Abb. 2:**
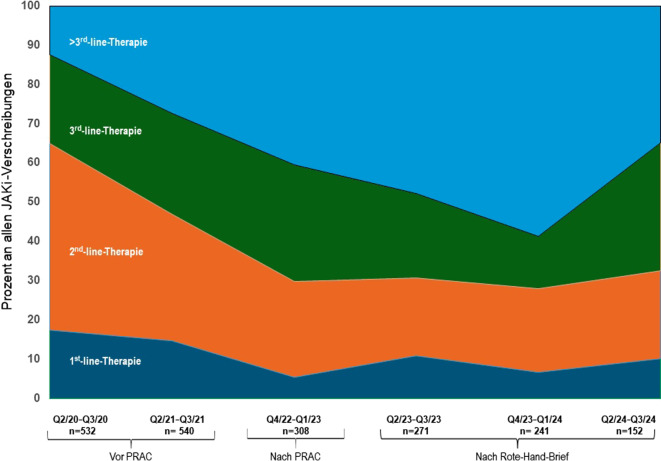
Veränderungen der Therapielinie, in der JAKi erstmalig verordnet wurden in Abhängigkeit von den Sicherheitsempfehlungen. *IL-6Ri* Interleukin-6-Rezeptor-Inhibitor, *JAKi* Januskinaseinhibitor, *PRAC* Pharmacovigilance Risk Assessment Comittee recommendations, *Q* Quartal, *TNFi* Tumornekrosefaktorinhibitor

Die Anzahl der Vortherapien bei JAKi-neu-behandelten Patient:innen nahm dabei deutlich zu und blieb deutlich höher als vor den regulatorischen Maßnahmen trotz Erholung der Neuverordnungszahlen (Tab. 2). Patient:innen erhielten im Verlauf deutlich seltener JAKi als Erstlinientherapie oder Zweitlinientherapie (Abb. 2). Die Anwendung als Zweitlinientherapie ging dabei am deutlichsten zurück (von 47,4 % in Q2/2020–Q3/2020 auf 20,0 % in Q2/2023–Q3/2023). Trotz wieder steigender Verordnungszahlen von JAKi kam es zu keinem Wiederanstieg des Einsatzes von JAKi als Erst- oder Zweitlinientherapie (Q2/2024–Q3/2024: 22,4 %). Konsekutiv zeigte sich ein starker Anstieg der Anwendung von JAKi als Viertlinienmedikament oder höher (Q2/2020–Q3/2020: 12,3 % vs. Q4/2023–Q1/2024: 58,7 %) (Abb. 2). Im letzten Halbjahr fand sich dann ein Rückgang von JAKi in der Viertlinientherapie zugunsten der dritten Linie, wobei der Einsatz in der ersten und zweiten Linie gleich blieb (Abb. 2).

**Tab. 2:** Patient:innencharakteristika von JAKi-Neubeginnern bei Therapiestart im Verlauf

Zeitraum	Alter (95 % CI)	Krankheitsdauer (95 % CI)	Vortherapien (95 % CI)
Q2/20–Q3/20	60,5 (58,3–62,7)	13,9 (12,2–15,5)	1,3 (1,2–1,5)
Q2/21–Q3/21	60,3 (58,7–62,0)	13,2 (11,9–14,5)	1,8 (1,7–2,0)
Q4/22–Q1/23	61,4 (58,7–64,2)	15,1 (12,7–17,4)	2,5 (2,2–2,8)
Q2/23–Q3/23	61,9 (58,8–65,1)	16,6 (13,6–19,5)	2,7 (2,2–3,2)
Q4/23–Q1/24	60,2 (57,1–63,3)	15,5 (13,3–17,6)	2,9 (2,5–3,3)
Q2/24–Q3/24	56.6 (53,4–59,8)	17,5 (14,6–20,5)	2,4 (1,9–2,9)

*95* *% CI* 95 %-Konfidenzintervall

## Differenzierter Einsatz von JAKis durch deutsche Rheumatolog:innen

Diese Follow-up-Analyse zu unserer Studie von 2024 zeigt damit, dass die JAKi-Neuverordnungen nach dem deutlichen Rückgang infolge der Veröffentlichung der Sicherheitsempfehlungen durch die PRAC bzw. die EMA aktuell wieder ansteigen, aber weiterhin kaum noch in den frühen Therapielinien wie zuvor eingesetzt werden [1].

Ein Rückgang der JAKi-Neuverordnungen infolge der Sicherheitsempfehlungen konnte auch in anderen Ländern wie den USA gezeigt werden [4]. Dass es zuletzt wieder zu einem Anstieg der Verordnungszahlen gekommen ist, mag auf eine differenziertere Betrachtung des Sachverhalts zurückzuführen sein: Zum einen wurden die Effekte in der ORAL Surveillance-Studie für den unselektiven JAKi Tofacitinib gezeigt [2]. Ergebnisse von randomisiert kontrollierten Sicherheitsstudien wie der ORAL Surveillance-Studie liegen bisher nicht für JAK 1-selektive JAKi vor [2]. Vorhandene Registeranalysen zeigen bisher kein konsistent erhöhtes Gesamtrisiko gegenüber bDMARD/TNFi [5, 6]. Zum anderen trat im Verlauf möglicherweise eine nüchterne Betrachtungsweise in den Vordergrund, denn die „number needed to harm“ (NNH) betrug in der ORAL Surveillance-Studie 550 RA-Patient:innen mit Tofacitinib, um ein MACE auszulösen, bzw. 276 für einen bösartigen Tumor [3]. Stellungnahmen und konkrete Behandlungsempfehlungen von Fachgesellschaften wie der DGRh haben zudem in der Folge möglicherweise zur Beruhigung beigetragen [3]. Es ist jedoch auf Basis unserer Daten zu vermuten, dass sich die verordnenden Rheumatolog:innen der Nebenwirkungen und Sicherheitsempfehlungen weiterhin bewusst sind und JAKi nur noch dann einsetzen, wenn andere Therapien nicht ausreichend wirksam waren. Dies zeigt sich an der Verwendung von JAKi größtenteils in der Drittlinie oder höher. Nach unserem Wissensstand sind diese Entwicklungen bisher noch nicht in anderen Ländern gezeigt worden. Eine leitliniengerechte Therapie kardiovaskulärer Risikofaktoren könnte weiter zu einer Reduktion des Risikos von JAKi beitragen [7].

Aufgrund der retrospektiven Datenerhebung ist die Dateninterpretation unserer Studie mit den typischen Einschränkungen solcher Studiendesigns behaftet. Weiterhin war es in unserer Analyse aufgrund der Art der Datenerhebung nicht möglich, den Zeitpunkt des Auftretens einer Komorbidität genau zu bestimmen, sodass nicht ermittelt werden konnte, ob das Auftreten von Neuerkrankungen Therapiewechsel zur Folge hatte und wir auf solche Analysen verzichten mussten. Andere Studien hatten in diesem Zusammenhang über rückläufige JAKi-Verordnungszahlen bei RA-Patient:innen mit den einschlägigen Risikofaktoren berichtet [4, 8]. Weiterhin bezieht unser Analysezeitraum die SARS-CoV-2-Pandemie (2020–2023) mit ein: In diesem Zeitraum kam es nachweislich zum einen zu Lieferengpässen bei bestimmten Biologika, wie z. B. dem IL-6-Ri Tocilizumab [9]. Andererseits kam es aber auch aufgrund von Sicherheitsbedenken in der Pandemie zum Rückgang von Neueinstellungen von Rituximab mit kompensatorischer Umstellung auf andere zielgerichtete Therapien [10]. Nach der Pandemie erfolgten dann eventuell auch wieder Rückumstellungen. Ob diese Effekte aber auf den relativen Anteil von JAKi an allen Verordnungen und die Therapielinie, in der JAKi verordnet wurden, einen relevanten Einfluss hatten, erscheint aber insgesamt unwahrscheinlich, sodass davon auszugehen ist, dass unsere Daten darlegen, dass es zu einem echten Wiederansteigen der Verordnungszahlen von JAKi in Deutschland gekommen ist. JAKi werden aber weiterhin im Vergleich zu den Zeiträumen vor den PRAC-Empfehlungen in den fortgeschrittenen Therapielinien eingesetzt, was auf einen sehr differenzierten Umgang mit dieser Substanzklasse bei deutschen Rheumatolog:innen hindeutet.

## Fazit für die Praxis

Im Anschluss an die Sicherheitsempfehlungen war ein deutlicher Rückgang der JAKi-Neuverordnungen zu beobachten. Inzwischen ist eine graduelle Zunahme der Verordnungszahlen erkennbar, wobei das Ausgangsniveau vor den Sicherheitsempfehlungen bislang nicht wieder erreicht wurde. JAKi werden weiterhin weniger in den frühen Therapielinien eingesetzt, was auf einen differenzierteren und zurückhaltenderen Umgang der verordnenden Rheumatolog:innen mit dieser Substanzklasse hinweist.

## Data Availability

Die Rohdaten dieser Studie sind nicht öffentlich zugänglich. Begründete Anfragen bezüglich der Rohdaten können an die Autoren gerichtet werden.
